# Fecal microbiota and metabolite profiles in patients with osteoarthritis: a cross-sectional study

**DOI:** 10.3389/fcimb.2026.1816705

**Published:** 2026-08-31

**Authors:** Qian-Qian Zhou, Jing Wang, Li-Lan Zhang, Ya-Ting He, Hao Liu, Yang-Guang Cao, Lin Cheng, Ya-Qin Zhang, Lei Zhang, Yi-Yuan Wang, Zhang-Wei Lu, Bao-Zhu Li

**Affiliations:** 1 Anhui Medical University, School of Public Health, Department of Epidemiology and Biostatistics, Center for Big Data and Population Health of IHM, Hefei, Anhui, China; 2 Research Center for Translational Medicine, The Second Affiliated Hospital of Anhui Medical University, Hefei, Anhui, China; 3 Department of Rheumatology and Immunology, The Second Affiliated Hospital of Anhui Medical University, Hefei, Anhui, China

**Keywords:** fecal metabolites, gastrointestinal microbiome, metabolomes, multiomics, osteoarthritis

## Abstract

**Introduction:**

Globally, Osteoarthritis (OA) represents a primary chronic joint condition, rooted in complex mechanisms such as metabolic irregularities, synovitis, immune system responses, and body-wide inflammation. Emerging evidence highlights gut microbiota as a critical mediator of joint inflammation.

**Methods:**

In this study, 55 OA patients from the affiliated hospital of Anhui Medical University were compared with 55 healthy controls. Using 16S rRNA (V4–V5) sequencing and POS/NEG dualionmode untargeted fecal metabolomics, we investigated the "gutmetabolismimmunityjoint" axis in OA.

**Results:**

The results indicate that patients with OA exhibit significant gut microbiota dysbiosis, characterised in particular by a reduction in beneficial bacterial groups such as *Clostridiales*, *Lactobacillales*, *Bacilli* and Actinobacteria, and an increase in *Bacteroidaceae* and *Burkholderiales*. Metabolomic analysis revealed that metabolic pathways such as beta-alanine metabolism and pyrimidine metabolism were enriched. Specific microbial changes are associated with alterations in host metabolism, for example, a reduction in the beneficial bacteria *Actinobacteria* was positively correlated with the downregulation of indole-3-acetic acid, which has a beneficial effect; *Bacilli* and *Lactobacillales* were associated with the dual loss of luteolin and D-tryptophan.

**Discussion:**

This study revealed significant alterations in gut microbiota and fecal metabolites in OA patients compared to healthy individuals. The identified differential metabolites hold promise as potential biomarkers for early diagnosis or therapeutic targets, offering new insights for future personalized interventions.

## 1 Introduction

Osteoarthritis (OA), as the most common chronic joint disease worldwide, poses a significant public health challenge (Hunter et al., 2020). In 2020, the condition affected approximately 7.6% of the global population, or 595 million people, and projections indicate that the number of patients will approach 1 billion by 2050 (Collaborator, GBD 2021 Gout, 2024). Clinically, OA is most prevalent among the elderly and is a leading cause of disability in older adults (Long et al., 2022). It is worth emphasizing that, although previous studies have largely focused on the elderly, data indicate that the incidence of OA peaks between the ages of 55 and 59 (Wang et al., 2025), suggesting that the disease is not exclusive to the very elderly; middle-aged individuals also face a significant risk. However, while most existing studies focus on obesity as a factor in the development of osteoarthritis (Whittaker et al., 2022), the risk of OA in non-obese individuals should not be overlooked. Animal studies have found that rats that received fecal microbiota transplants from non-obese OA patients exhibited even more severe cartilage degeneration and synovitis than those that received transplants from obese patients; population-based studies have also shown that, compared with individuals who have consistently maintained a normal weight, those who were previously obese but later lost weight to a non-obese state still have a 34% higher risk of OA (Cui et al., 2024). A growing body of evidence suggests that the risk of osteoarthritis remains significant among non-obese individuals. This highlights the importance of focusing on the risk of OA in this population. Therefore, increasing awareness of OA and strengthening attention to middle-aged groups holds significant clinical and public health implications.

Traditionally, OA has been considered primarily caused by mechanical stress and aging, with early research focusing largely on the degeneration of articular cartilage, subchondral bone remodeling, and changes in the extracellular matrix. However, recent studies have revealed that systemic inflammation, synovitis, metabolic dysregulation, and immune cell activation play key roles in the disease process (Chow and Chin, 2020; Batarfi et al., 2025), shifting the understanding of OA from a purely mechanical condition to a multifactorial disease with complex metabolic and immune underpinnings. Against this backdrop, the “gut-joint axis” theory has been proposed, suggesting that dysbiosis may influence joint degeneration through metabolic and inflammatory signaling pathways (You et al., 2025; Zhang et al., 2024). Existing reviews have indicated that alterations in specific metabolites in blood, urine, and synovial fluid samples may serve as potential biomarkers for predicting the onset and progression of OA (Liao et al., 2023).Furthermore, through a combined analysis of gut microbiota and serum metabolites, Wang et al. (Wang et al., 2025) were the first to reveal the potential role of lipid metabolism disorders in the progression of OA. This suggests that identifying changes in specific gut microbiota and metabolites is of great significance for studying the pathogenesis and progression of OA. It is worth noting that there are certain differences in research approaches regarding the combined application of gut microbiota and metabolomics. Taking rheumatoid arthritis (RA) as an example, some studies have integrated gut microbiota and fecal metabolomics, revealing correlations between gut microbiota and metabolites (Yu et al., 2021), while other studies have focused on changes in serum metabolite levels to investigate the pathogenesis and progression of RA (Cai et al., 2024). These two approaches have distinct focuses: serum metabolomics is better suited for capturing systemic metabolic reprogramming, whereas fecal metabolomics can more directly and accurately reflect the metabolic output of the gut microbiota and its immediate impact on the intestinal microenvironment. However, current research in the field of OA has largely focused on the elderly population, many of whom have metabolic confounders such as obesity. Regarding non-obese (normal BMI) middle-aged OA patients, our understanding of the association between gut microbiota and fecal metabolites has not been extensively characterized, and systematic exploration of their interactions and underlying mechanisms remains unexplored. Therefore, systematically incorporating the analysis of fecal metabolites and gut microbiota in non-obese middle-aged OA populations holds significant scientific value.

To this end, further examining the associations between the gut microbiota and fecal metabolites will contribute to a more comprehensive description of changes in the gut microenvironment during OA progression. Fecal metabolites provide a more direct reflection of microbial metabolic activity and its effects on host inflammation and energy metabolism, and thus have greater functional relevance than analyses of microbial community structure alone. Although existing evidence suggests a link between gut microbiota dysbiosis and OA, and systematic analyses of fecal metabolites in OA patients have been reported, the synergistic influence of dysbiosis and metabolic profile alterations on joint degeneration warrants further investigation. Thus, we propose the following research hypothesis: the composition of the gut microbiota and the fecal metabolite profile in patients with osteoarthritis are significantly altered compared to healthy controls, and that these alterations are closely associated with each other, potentially reflecting a state of host–microbiome metabolic dysregulation that may be related to OA pathogenesis.

Based on this, the present study aims to systematically elucidate the multidimensional interactions between the gut microbiota and fecal metabolic activity in patients with OA, and to construct a “microbiota-metabolite-joint inflammation” network. Previous research on OA has emphasized blood metabolites. The present study extends this line of investigation to fecal metabolites in middle-aged, non-obese individuals, providing a systematic analysis and offering new insights into pathogenesis and treatment strategies.

## 2 Materials and methods

### 2.1 Study individuals and sample collection

This study employed a cross-sectional design, enrolling patients with OA attending the Department of Orthopaedics at the Affiliated Hospital of Anhui Medical University to form the OA group. Additionally, healthy volunteers matched 1:1 for age, sex and body mass index were recruited from the community to form the healthy control group (HCs). All participants were excluded if they had a history of cardiovascular disease, malignant tumours, autoimmune diseases or active infectious diseases. Inclusion criteria for the OA group were further restricted to: ① no metabolic diseases such as obesity or diabetes; ② no use of antibiotics or probiotic individuals within 3 months prior to enrolment; ③ no history of malignant tumours, no severe cardiac, hepatic or renal insufficiency, and no organic gastrointestinal diseases; ④ no concomitant autoimmune diseases. Inclusion criteria for the HC group included: ① no history or family history of OA or other chronic diseases; ② no severe organic diseases, cardiovascular diseases, metabolic disorders or autoimmune diseases; ③ voluntary participation in the study. This study protocol was approved by the Ethics Committee (No.YX2024-243 (F1)), and all participants signed a written informed consent form. The study aimed to analyse differences in gut microbiota structure and faecal metabolome profiles (dependent variables) between two independent groups (OA vs. HCs), while adjusting for potential confounders including BMI, age, sex, and dietary habits. All participants collected fresh faecal samples in the early morning; following centrifugation, these were immediately stored at -80 °C for subsequent testing and analysis.

For all enrolled patients, the baseline characteristics are presented in **Table 1**. There were no statistically significant differences between the two groups in terms of age, weight, height, gender and BMI (*P* > 0.05) (**Table 1**). As shown in **Supplementary Table 1**, there were no statistically significant differences in the intake of 23 dietary nutrients between the two groups.

**Table 1:** Basic information of the OA case group and control group.

Characteristic	OA (n=55)	HCs (n=55)	*P*-value
Age (years)	58.80 ± 7.37	57.45 ± 5.25	0.470
Height (cm)	157.21 ± 31.22	165.22 ± 8.26	0.079
Weight (kg)	60.60 ± 10.89	63.07 ± 9.36	0.311
Male	15 (27.3%)	15 (27.3%)	1.000
Female	40 (72.7%)	40 (72.7%)	
BMI (kg/m²)	22.64 ± 3.46	22.89 ± 1.85	0.820

BMI, Body Mass Index.

### 2.2 16S rRNA microbiota analysis

To assess compositional differences in microbial communities, we analysed their diversity using high-throughput 16S rRNA gene sequencing technology. Following Illumina paired-end sequencing, the initial data processing steps included demultiplexing and stringent quality control. The reads were subsequently assembled and filtered to eliminate errors and artifacts, ensuring the robustness of the data for downstream analysis. For taxonomic profiling, sequences were clustered into OTUs at a 97% identity cutoff, and their classification was inferred by comparison with a curated database. The ecological structure of the communities was interrogated through a suite of diversity indices and pairwise distance measures. Multivariate statistical methods, including principal coordinates analysis, were applied to visualize sample clustering. To assess the significance of community differences across groups, we applied permutation tests along with biomarker discovery algorithms. Furthermore, Linear Discriminant Analysis Effect Size (LEfSe) was used to identify various features in order to investigate biomarkers that showed significant differences between the case group and the control group at different levels. The Kruskal-Wallis H test to identify gut microorganisms with significant differences in abundance distribution across different groups (*P* < 0.05). Based on this, Linear Discriminant Analysis (LDA) scores were then used to evaluate each microbial community. In this study, the default LDA score threshold was set to 2; species with absolute scores exceeding 2 were considered significantly enriched (http://huttenhower.sph.harvard.edu/galaxy/). In addition, this study used the PICRUSt2 (v2.5.2) software to predict functional genes from 16S rRNA sequencing data (Douglas et al., 2020).

### 2.3 LC-MS metabolomics analysis

Liquid chromatography-mass spectrometry (LC-MS) data were processed using MS-DIAL software, with steps including peak identification, filtering, alignment and compound identification, ultimately yielding a data matrix containing m/z, retention time and peak area. Mass spectrometry parameters: in positive ion mode, electrospray voltage 3.0 kV, S-Lens RF 30%; in negative ion mode, 3.2 kV, S-Lens RF 60%. Heater 300 °C, capillary 350 °C, sheath gas/auxiliary gas/tail gas 45/15/1 arb. Full scan m/z 70–1050 (resolution 70,000), with automatic triggering of HCD secondary scans for the top 10 parent ions (resolution 17,500).

### 2.4 Association analysis

The faecal metabolomics analysis in this study primarily involved merging data obtained from the positive (POS) and negative (NEG) ion modes after separate processing, and exporting them for subsequent analysis. The data were then subjected to orthogonal partial least squares discriminant analysis (OPLS-DA). Differentially expressed metabolites were screened using *P*-values obtained from univariate analysis and variable importance in projection (VIP) values from the OPLS-DA analysis. The screening criteria were a *P*-value of less than 0.05 and a VIP value greater than 1. Furthermore, Spearman’s correlation coefficient was used to analyse the correlations between the various differentially expressed metabolites. Finally, to elucidate the functions of the differentially expressed metabolites from a systems biology perspective, the list of selected differentially expressed metabolites was mapped to the Kyoto Encyclopaedia of Genes and Genomes (KEGG) database for pathway enrichment analysis. Enrichment analysis was performed using hypergeometric distribution tests to calculate *P*-values, thereby identifying sets of metabolites enriched in specific metabolic pathways.

Spearman correlation analysis was performed to link differential microbial taxa (identified by LEfSe across all taxonomic levels) with significantly altered metabolites (from MS2 data, selected by *p* < 0.05 and VIP > 1). To control for false discovery rate (FDR) due to multiple comparisons, all correlation *P*-values were adjusted using the Benjamini-Hochberg method. Only correlation pairs with an adjusted *P* < 0.05 (*q* < 0.05) were retained as statistically significant. The remaining significant associations were plotted as a heatmap using ggplot2, where point colours denote the direction and strength of the Spearman’s correlation coefficient.

### 2.5 Machine learning

To further identify and distinguish the most informative metabolites from osteoarthritis patients across two distinct clinical centres, a random forest (RF) model was constructed using the MS2-POS dataset. Patients from each hospital were randomly partitioned into training (70%) and testing (30%) cohorts to assess model robustness. Variable importance was evaluated using dual metrics: mean accuracy reduction and mean Gini index reduction.

## 3 Results

### 3.1 Identification of the signatures of the gut microbiota profile and function

**Figure 1:**
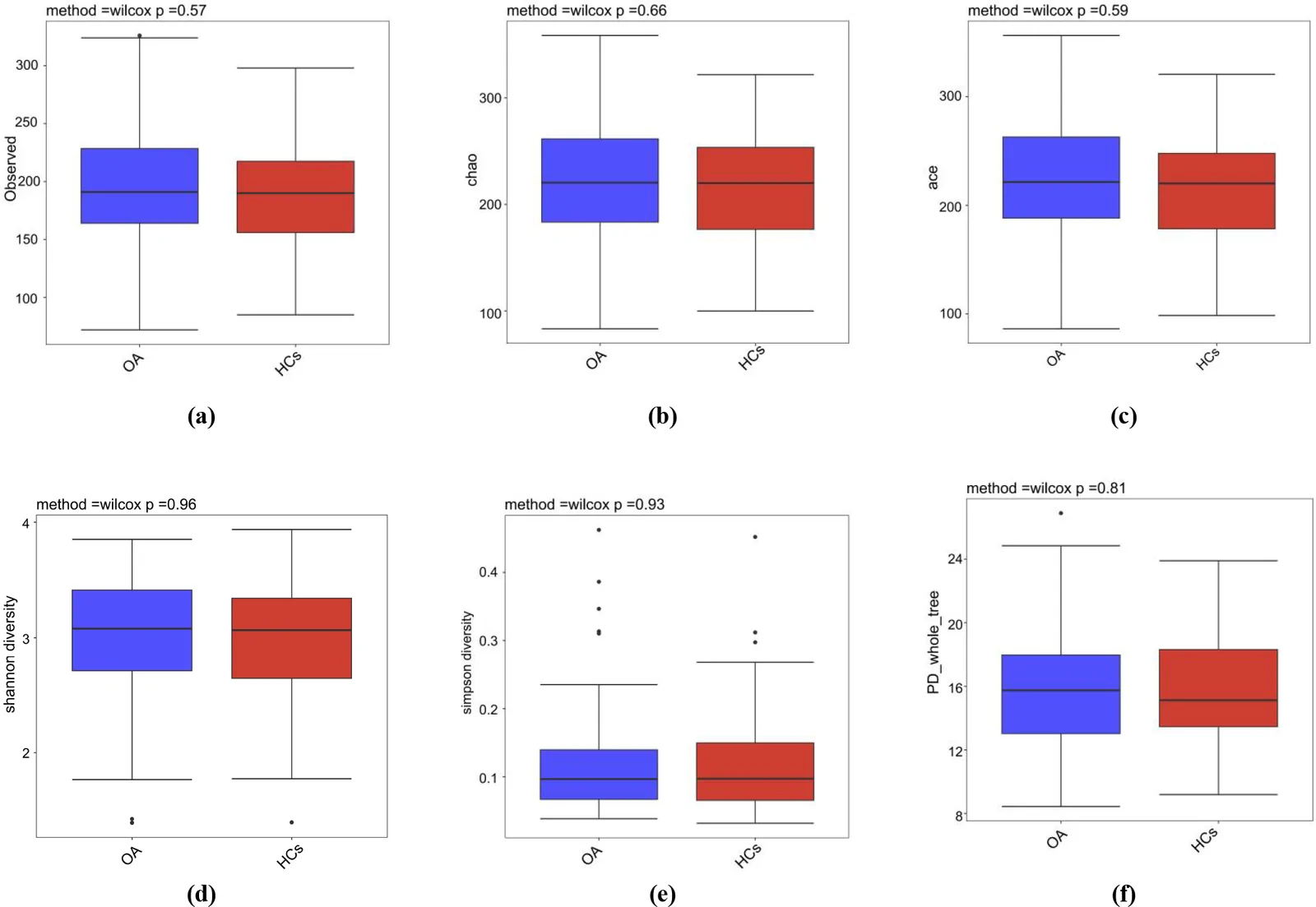
Alpha diversity analysis of gut microbiota between the two groups: **(a)** Observed species; **(b)** chao-1; **(c)** ace; **(d)** shannon; **(e)** simpson; **(f)** PD_whole_tree.

**Figure 2:**
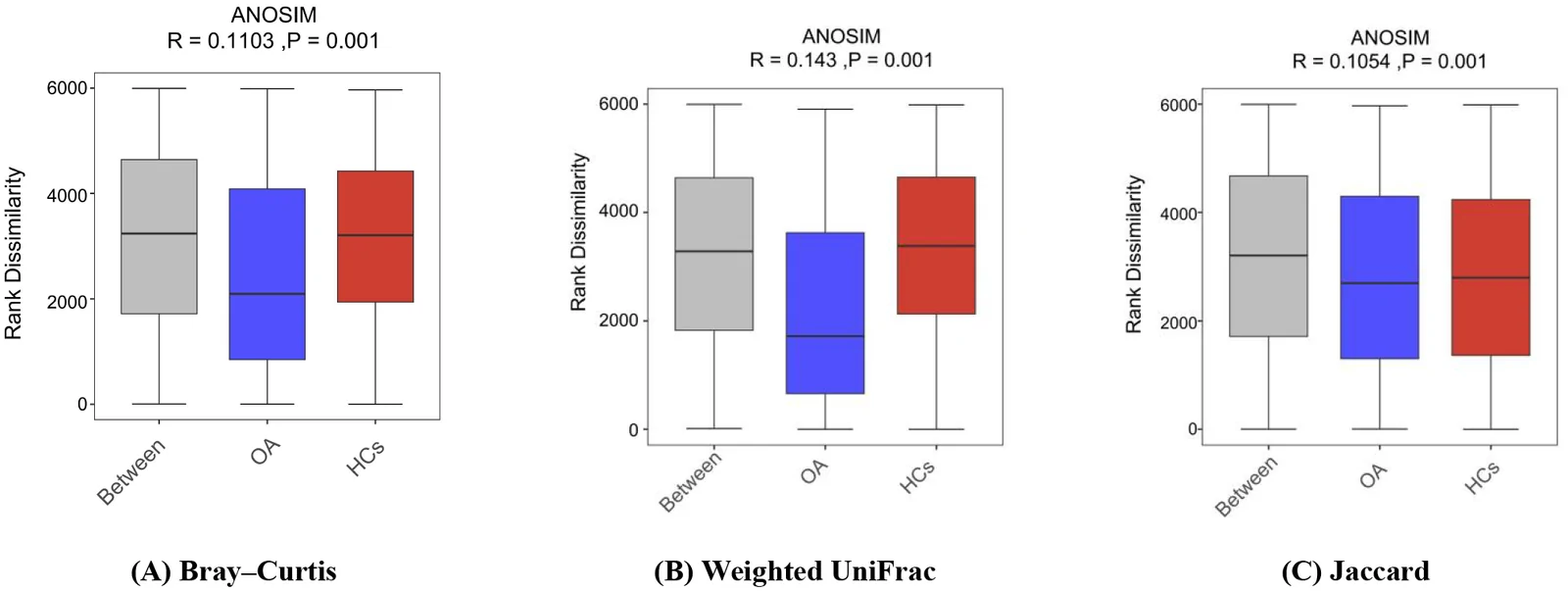
ANOSIM boxplots comparing beta diversity between the two groups of gut microbiota, based on three distance metrics **(A)** Bray–Curtis; **(B)** Weighted UniFrac; **(C)** Jaccard.

The results of the alpha diversity analysis showed that, following comparison via the Wilcoxon rank-sum test (**Figure 1**), the OA group and the HCs group exhibited similar values for all indicators reflecting species richness (Observed species, Chao-1 and ACE indices); similarly, no statistically significant differences were observed between the groups regarding the Shannon and Simpson indices, which reflect community evenness and overall diversity. Furthermore, no significant differences were observed between the two groups in the PD_whole_tree index, which is used to assess phylogenetic diversity within the community (*P* = 0.81). Beta diversity analysis was performed using the ANOSIM test (**Figure 2**), and the results indicated a statistically significant difference in the overall community structure of the gut microbiota between the OA group and the HCs group, suggesting that the microbial community composition of patients with osteoarthritis has undergone significant changes at the macrostructural level. The NMDS analysis was consistent with the findings of the ANOSIM test, visually illustrating the overall trend of separation in microbial community structure between the groups (**Supplementary Figure 1**). PCoA analysis further revealed that, although the 95% confidence ellipses of the OA group and the HCs group overlapped partially, the centres of sample distribution and the directions of dispersion exhibited a clear separation between the two groups (**Supplementary Figure 2**).

**Figure 3:**
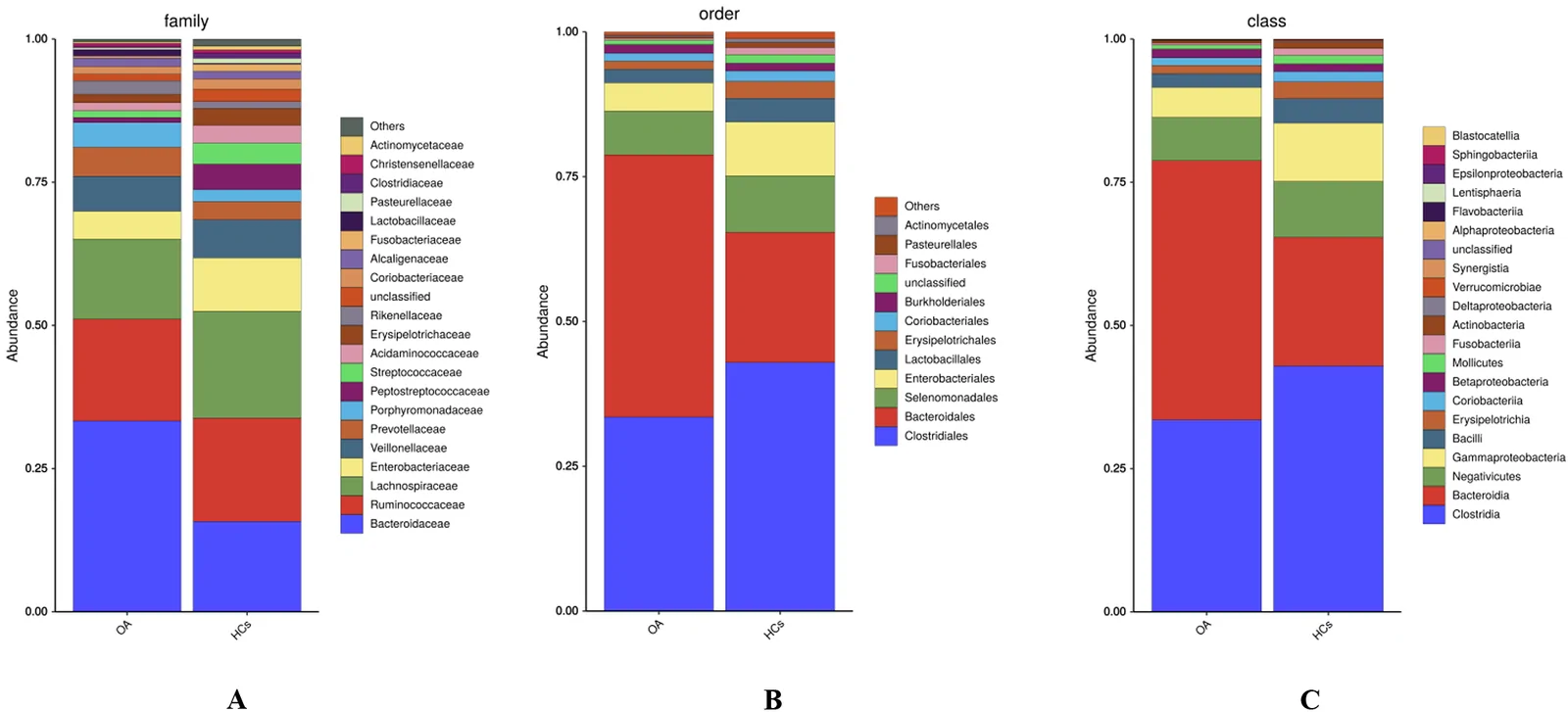
Gut microbiota composition in the two groups at family **(A)**, order **(B)**, class **(C)** levels, respectively.

At the family level, the most abundant bacterial families in both sets of samples included: *Bacteroidaceae*, *Ruminococcaceae*, *Lachnospiraceae*, *Enterobacteriaceae*, *Veillonellaceae*, *Prevotellaceae*, *Porphyromonadaceae*, *Peptostreptococcaceae*, *Streptococcaceae*, *Acidaminococcaceae*, *Erysipelotrichaceae* and *Rikenellaceae* (**Figure 3A**). Among these, *Bacteroidaceae*, *Porphyromonadaceae* and *Ruminococcaceae* exhibited higher relative abundances in the OA group than in the HCs group, whilst *Enterobacteriaceae* and *Peptostreptococcaceae* exhibited higher relative abundances in the HCs group than in the OA group(**Supplementary Figure 3A**). At the order level, the leading bacterial families in terms of relative abundance in both sample groups included *Clostridiales*, *Bacteroidales*, *Selenomonadales*, *Enterobacteriales*, *Lactobacillales*, *Erysipelotrichales*, *Corynebacteriales*, *Burkholderiales*, *unclassified*, *Fusobacteriales*, *Pasteurellales* and *Actinomycetales* (**Figure 3B**). Among these, *Clostridiales*, *Lactobacillales* and *Enterobacteriales* were reduced in the OA group, whilst *Bacteroidales* and *Burkholderiales* were increased in the OA group (**Supplementary Figure 3B**). At the class level, the major bacterial families with the highest relative abundances in both sample groups included *Clostridia*, *Bacteroidia*, *Mollicutes*, *Gammaproteobacteria*, *Bacilli*, *Erysipelotrichia*, *Coriobacteriia*, *Betaproteobacteria*, *Mollicutes*, *Fusobacteriia* and *Actinobacteria*, with *Clostridia* (**Figure 3C**), *Bacilli* and *Actinobacteria* showing a decline in the OA group, whilst *Bacteroidia* showed an increase in the OA group (**Supplementary Figure 3C**). Additionally, PICRUSt2 functional prediction analysis identified the glycan biosynthesis and metabolism and transport and catabolism pathways as the most significantly upregulated, and the membrane transport pathway as the most significantly downregulated, among all predicted functional categories in the gut microbiota of OA patients (**Supplementary Figure 4**).

**Figure 4:**
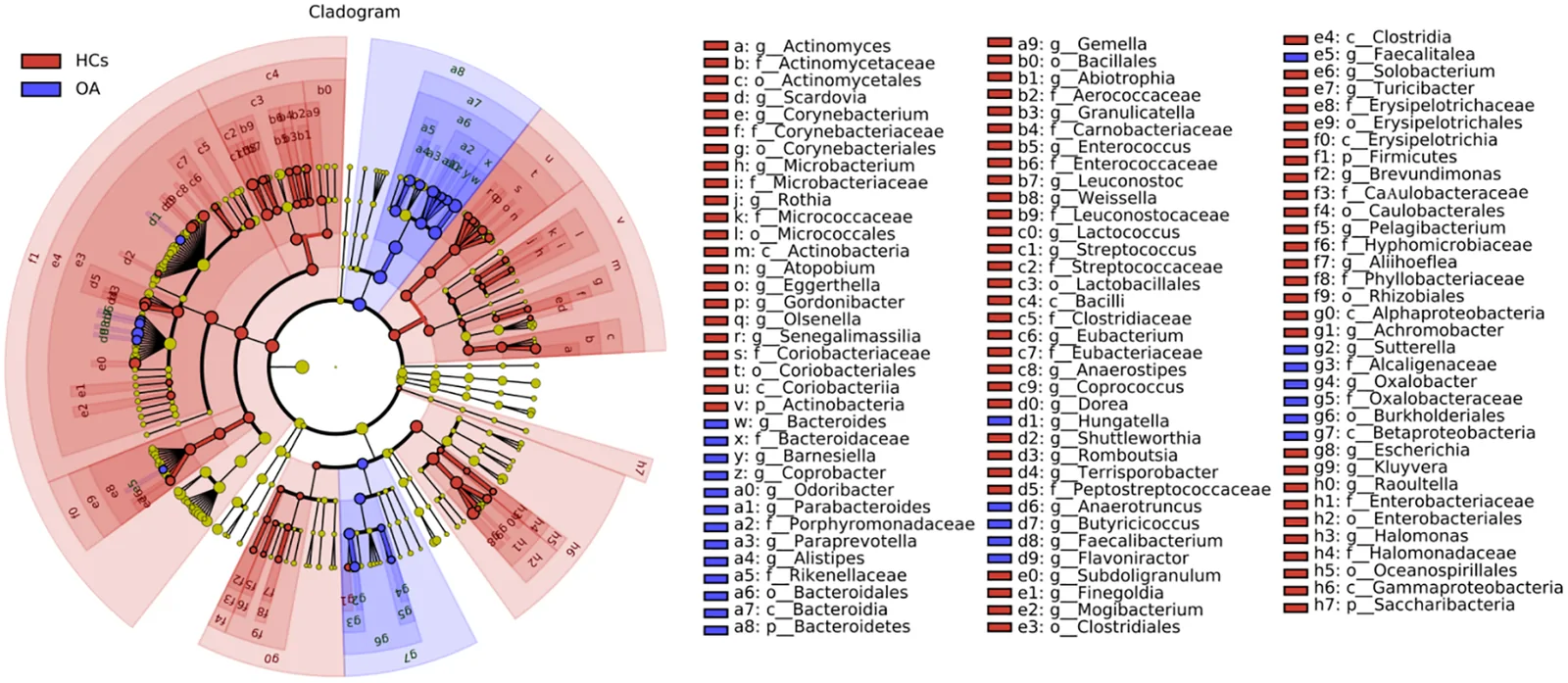
Differential species distribution map between the OA group and HCs group obtained through LEfSe analysis.

LEfSe was used to screen for differential microbial communities between the OA group and the healthy control (HCs) group, and this analysis identified multiple taxa with significant LDA scores, highlighting unique microbial characteristics between the two groups (**Supplementary Figure 5**). The cladogram revealed distinct dominant lineages in the two groups (**Figure 4**): the OA group was enriched with microbial lineages primarily centred on *Bacteroides uniformis*, *Bacteroides fragilis*, and others; In contrast, the HCs group was enriched with bacterial communities primarily comprising *Actinobacteria*-*related lineages*, *Clostridiales*, several lineages of *Lactobacillales*, and *Enterobacteriaceae*.

### 3.2 Revealing alterations in distinct metabolites in OA patients via the fecal metabolome

**Figure 5:**
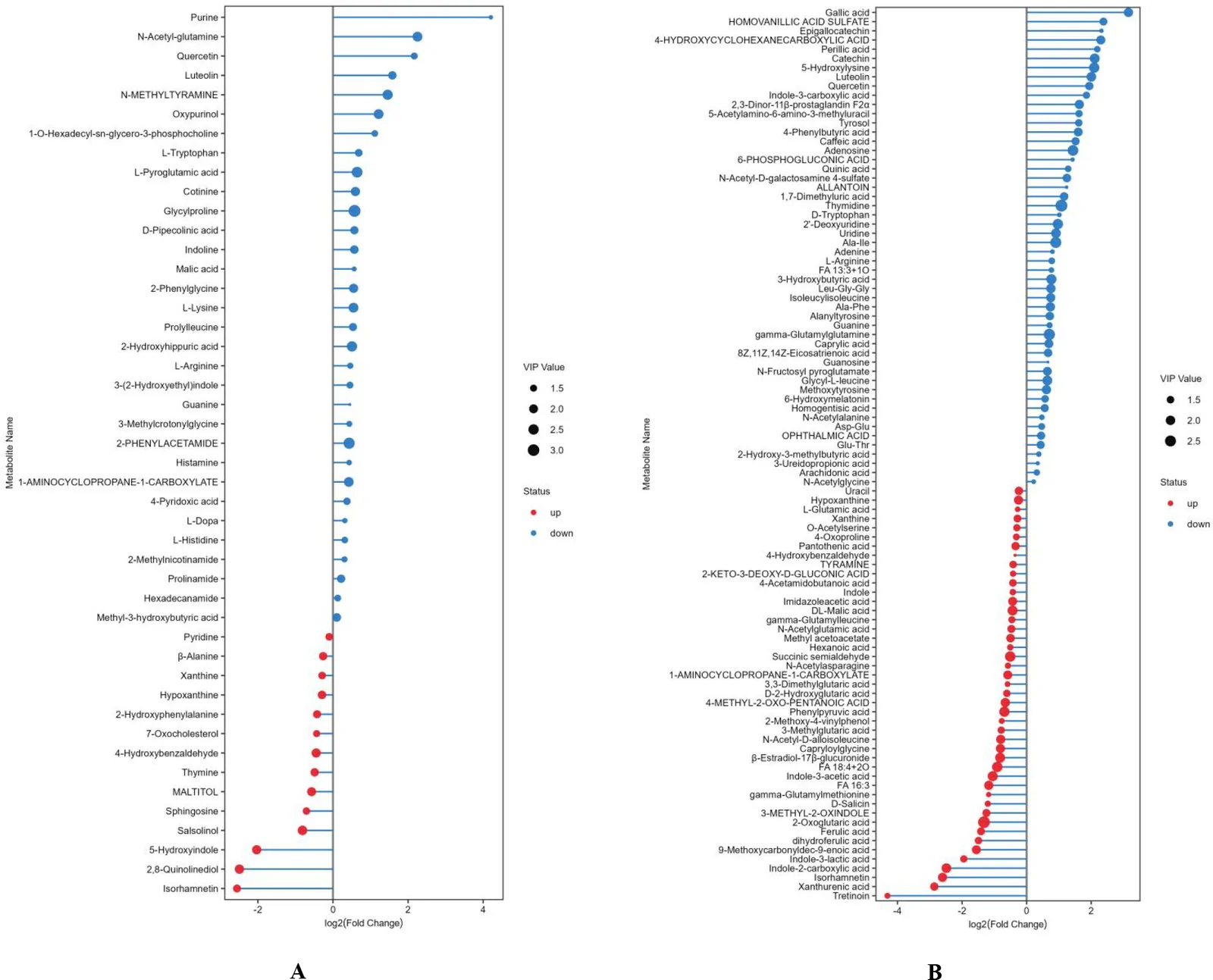
Stem diagram indicated the different metabolites between two groups in POS mode (VIP >1) **(A)**; Stem diagram indicated the different metabolites between two groups in NEG mode (VIP >1) **(B)**.

As illustrated, the unsupervised PCA analysis yielded a scatter plot showing cross-clustering between comparison groups, indicating data stability and reliability (**Supplementary Figures 6A, B**). OPLS-DA analysis revealed distinct quadrant distributions in the comparison group scatter plot, signifying significant differences in metabolic patterns (**Supplementary Figures 6C, D**). We identified differentially expressed metabolites using the criteria of VIP ≥ 1 and *P* < 0.05, which included both upregulated and downregulated species, all reaching significance (*P* < 0.05 and FDR < 0.05). Subsequently, we found that 46 pairs and 97 pairs of differentially expressed metabolites were highly correlated (R = 1) in POS and NEG modes, respectively (**Supplementary Figure 7**). Based on secondary mass spectrometry (MS2) identification, we identified differentially expressed metabolites in both POS and NEG modes. We presented the top three metabolites with the smallest FDR-adjusted *P* values in each direction. In POS mode, a total of 46 differentially expressed metabolites were identified (**Figure 5A**) of which 14 were up-regulated in the OA group compared to the HCs group (2,8-Quinolinediol, Isorhamnetin, MALTITOL, etc.), whilst 32 were downregulated (N-Acetyl-glutamine, Oxypurinol, Glycylproline, etc.), the complete list is provided in **Supplementary Table 2**. A total of 97 differentially expressed metabolites were identified in the NEG mode(**Figure 5B**), of which 52 were up-regulated in the OA group compared to the HCs group (Indole-2-carboxylic acid, Isorhamnetin, 9-Methoxycarbonyldec-9-enoic acid, etc.), whilst 45 were downregulated (Leu-Gly-Gly, Ala-Ile, Isoleucylisoleucine, etc.), the complete list is provided in **Supplementary Table 3**.

### 3.3 Revealing functional pathways associated with differentially expressed fecal metabolites

**Figure 6:**
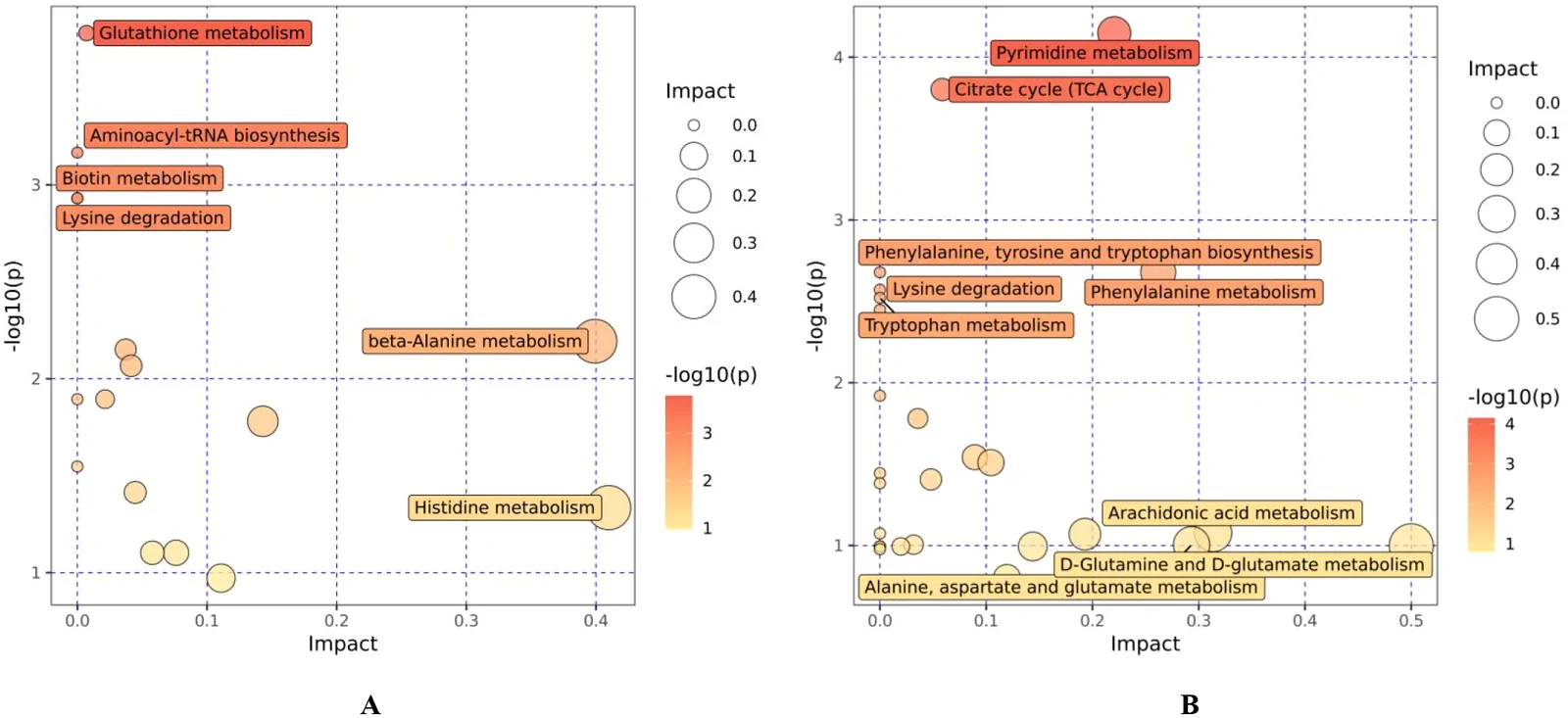
Metabolic pathway diagram in POS mode **(A)** and Metabolic pathway diagram in NEG mode **(B)**.

This study selected differentially expressed metabolites identified via the POS model (VIP > 1 and *P* < 0.05) and mapped them to the KEGG database for pathway enrichment and pathway impact analysis (**Figure 6A**). The results indicate that multiple pathways show signs of disruption in terms of statistical significance (−log_10_*P*) and pathway impact; pathways such as aminoacyl-tRNA biosynthesis and lysine degradation appear to be relatively enriched. Topological analysis further reveals that beta-alanine metabolism has a high pathway impact value. In NEG mode, KEGG pathway enrichment and topological analysis were also performed on differentially expressed metabolites. The results showed that pyrimidine metabolism was significantly altered; in addition, the citrate cycle, TCA cycle, alanine, aspartate, and glutamate metabolism, and Arachidonic acid metabolism were also significantly enriched (**Figure 6B**). At the same time, we conducted a further analysis of the upregulated and downregulated metabolites and found that pathways such as beta-Alanine metabolism, Phenylalanine metabolism were significantly enriched among the upregulated metabolites, while pathways such as Pyrimidine metabolism were significantly enriched among the downregulated metabolites (**Supplementary Figure 8**).

### 3.4 Analysis of the correlation between differentially expressed gut microbiota and differentially expressed metabolites

Based on the differentially expressed metabolites identified through screening—those that were significantly up-regulated or down-regulated in the OA group compared with the HCs group—we conducted a further analysis of the association between the gut microbiota and faecal metabolomics. The results showed that, among the significantly correlated metabolite–microbiota pairs, we selected those with the strongest correlation coefficients for detailed presentation (**Supplementary Table 4**). Specifically, the abundance of *Bacteroidaceae*, which was increased in OA patients, showed a significant positive correlation with 2-oxoglutaric acid in faecal metabolites, a metabolite that was also upregulated in the OA group. Additionally, the decreased abundance of *Clostridiales* in OA patients was positively correlated with the downregulation of Isoleucylisoleucine, which was also downregulated in OA. Furthermore, 1-aminocyclopropane-1-carboxylate exhibited a positive correlation with *Bacteroidaceae* in NEG mode, whereas no significant correlation was observed in POS mode. The complete list of all significant correlation pairs is available in **Supplementary Figure 9**.

### 3.5 Selection of key metabolites based on machine learning

**Figure 7:**
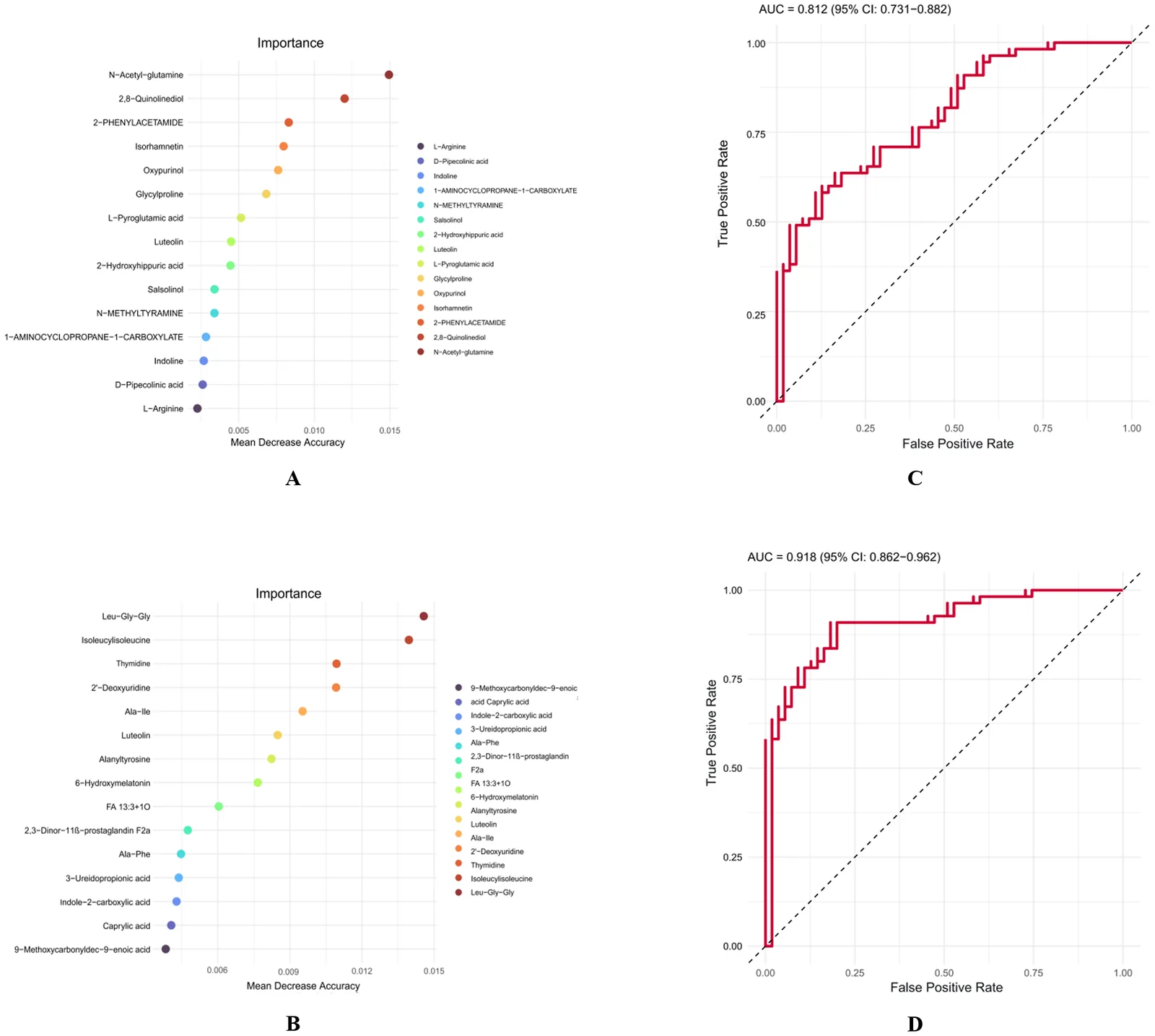
Identification and importance ranking of key differential metabolites by random forest model, and ROC curve analysis for predictive performance. POS mode: feature importance ranking of key differential metabolites (Mean Decrease Accuracy) **(A)**; NEG mode: feature importance ranking of key differential metabolites (Mean Decrease Accuracy) **(B)**; ROC curves for the POS-derived metabolite panel in the discovery and validation sets (AUC with 95% CI) **(C)**; ROC curves for the NEG-derived metabolite panel in the discovery and validation sets (AUC with 95% CI) **(D)**.

Using random forest machine learning, we conducted a series of analyses on the total differentially expressed metabolites. The results indicated that N-Acetyl-glutamine and Leu-Gly-Gly were key metabolites in both the POS and NEG patterns (**Figure 7**), demonstrating good predictive performance. To further explore upregulated and downregulated metabolites, we identified isorhamnetin and 2,8-quinolinediol as key metabolites in the upregulated group (**Supplementary Figure 10**), while 2’-deoxyuridine and N-acetylglutamine were key metabolites in the downregulated group(**Supplementary Figure 11**). These findings indicate that machine learning-based feature selection can effectively screen for candidate biomarkers, further confirming the significance of specific fecal metabolites as reliable indicators for stratifying osteoarthritis across different clinical populations.

## 4 Discussion

Firstly, this study conducted a systematic analysis of the diversity characteristics of the gut microbiota. The results showed that, whilst there were no significant changes in the alpha diversity of the gut microbiota in patients with OA, this finding is consistent with the trends in microbial structural changes in OA patients reported by Wang et al (Wang et al., 2023). A significant structural shift was observed at the beta diversity level. This suggests that the overall ecological structure of the microbiota undergoes a remodelling in the disease state, rather than merely an increase or decrease in species abundance. This study found an upward trend in *Bacteroidaceae* in the OA group. This finding differs from previous reports of a downward trend in *Bacteroidaceae* in obese OA patients (Schott et al., 2018). This seemingly contradictory phenomenon may not be coincidental, but rather reflects the microbial heterogeneity among populations with different metabolic backgrounds. Previous reviews have indicated that the abundance of *Bacteroidaceae* is typically lower in obese individuals than in non-obese individuals (Pinart et al., 2021), whereas the cohort included in this study comprised non-obese patients. Furthermore, studies have also observed an increase in the *Bacteroidota, Bacteroides* among OA patient (Liu et al., 2022), further suggesting that changes in specific bacterial species may be population-specific. From a functional mechanism perspective, Schirmer et al. (Schirmer et al., 2018) suggested that the expansion of *Bacteroidetes-associated microbiota* may be closely associated with gut microbiota dysbiosis and abnormal activation of the host immune system. Strains such as *Bacteroides fragilis* can directly regulate the activity of immune cells via lipopolysaccharide (LPS), a component of their cell walls, thereby activating T cells, B cells and macrophages (Schirmer et al., 2018; Mancuso et al., 2005). LPS can enter the systemic circulation via a compromised intestinal barrier, subsequently migrating to the local joint microenvironment, where it binds to corresponding receptors on the surface of synovial macrophages and chondrocytes, triggering the release of pro-inflammatory factors such as TNF-α, IL-1β and IL-6, thereby accelerating the degradation of the cartilage matrix and the destruction of joint structures (Hashizume et al., 2023). The aforementioned cascade of immune activation can induce and sustain a state of chronic low-grade inflammation, which is widely recognised as one of the core pathological mechanisms underlying the onset and progression of OA (Terkawi et al., 2022). *Ruminococcaceae* also showed an upward trend in the OA group in this study. Given that the direction of change for this family in OA remains controversial in the existing literature—with some studies reporting increased abundance (Xu et al., 2022) and others observing a decrease (Wang et al., 2023), this difference may suggest that fluctuations in their abundance are jointly regulated by the stage of the disease, the host’s metabolic state, and functional heterogeneity within the bacterial genus. Furthermore, this study found that the relative abundance of *Porphyromonadaceae* was higher in the OA group than in the control group. Although this family has not yet been definitively identified as a core differential family in knee OA, it has been reported to show a significant increase in abundance in spondyloarthropathies such as ankylosing spondylitis, and this increase is associated with disease severity (Costello et al., 2015), suggesting that ‘pathogenic microbiota expansion’ may be a common pathological feature across different arthritis phenotypes (Zhao et al., 2018).The increased abundance of *Burkholderiales* in the OA group is consistent with the findings of Ning et al. in OA patients (Ning et al., 2022), further validating the trend towards enrichment of this order in OA. However, Li et al. observed a significant reduction in its abundance in a mouse model of osteoporosis, which was positively correlated with bone formation markers and intestinal barrier function (Li et al., 2024). This contrast suggests that the elevation of *Burkholderiales* in OA may exert dual effects, both pro-inflammatory and osteogenic, providing a new perspective on understanding the complex role of the gut microbiota in the abnormal bone metabolism associated with OA. Previous studies have reported a reduction in specific beneficial bacteria, such as *Bacteroides plebeius* and *Faecalibacterium prausnitzii*, in patients with OA (Wang et al., 2025). Building on these findings, this study observed that the relative abundance of beneficial bacterial groups, including *Clostridiales*, *Lactobacillales*, *Bacilli*, and *Actinobacteria*, was also significantly lower in the OA group compared to the healthy control group. *Clostridiales* and *Lactobacillales* are the primary producers of short-chain fatty acids in the gut; their reduction can directly impair intestinal barrier function and exacerbate systemic low-grade inflammation (Sun et al., 2025), thereby accelerating cartilage degeneration. The reduction in *Actinobacteria* and *Lactobacillus*, meanwhile, implies a weakening of microbiota-mediated immune regulation and anti-inflammatory capacity (Song et al., 2025). Crucially, Mendelian randomisation studies have confirmed a negative causal relationship between *Actinobacteria* abundance and the risk of OA onset (Li et al., 2024), providing causal evidence for their protective role. Lan et al. also observed significant changes in *Bacilli* abundance in rats with OA (Lan et al., 2021), supporting the association between this family and the pathological state of OA. Furthermore, in this study, the abundance of *Enterobacteriaceae* and *Peptostreptococcaceae* showed a downward trend in non-obese OA patients, in stark contrast to previous reports of increased abundance of these genera in obesity-associated OA (Schott et al., 2018). This discrepancy suggests that obesity-related metabolic disorders may be a key driver of the aforementioned microbial changes, highlighting the value of stratification based on metabolic background in studies of the gut microbiota in OA.

Based on non-targeted faecal metabolomics analysis, levels of xanthine and hypoxanthine were significantly elevated in the OA group under the POS model, suggesting abnormal purine metabolism and exacerbated intra-articular oxidative stress in OA; ROS generated by the key enzyme XOR may exacerbate oxidative damage to cartilage and pathological calcification (Nasi et al., 2021). Furthermore, the tryptophan metabolite 5-hydroxyindole was also upregulated. Previous studies have confirmed that tryptophan metabolism is disrupted in OA, mediated by gut microbiota dysbiosis (Wei et al., 2023), suggesting that the microbial metabolic pathways for tryptophan undergo significant alterations in the OA state. Furthermore, levels of 2-hydroxyphenylalanine, a hydroxylated derivative of phenylalanine, were also elevated. Phenylalanine has been proposed as a plasma biomarker for the progression of knee OA (Zhai et al., 2021), and the elevation of its derivatives further suggests that phenylalanine metabolic disorders are involved in the pathological process of OA (Ge et al., 2025). Concurrently, several key amino acids—including L-lysine, L-arginine, L-tryptophan and L-histidine—and their derivatives showed a downregulated trend. L-lysine and L-arginine are essential for cartilage matrix synthesis and skeletal health (de Paz-Lugo et al., 2023; Aziz et al., 2024); their downregulation suggests insufficient collagen synthesis, which may be a metabolic reflection of cartilage degeneration. Furthermore, 2-hydroxyhippuric acid derivatives possess antioxidant, anti-inflammatory and bone resorption-inhibiting properties (Chen et al., 2021); their downregulation may have an adverse effect on OA. Indoline derivatives can delay the progression of OA by inhibiting the NF-κB and ERK pathways (Zhu et al., 2021); their downregulation suggests a reduction in endogenous protective factors, making pro-inflammatory pathways more susceptible to activation. In the NEG model, levels of 2-oxoglutaric acid and DL-malic acid—key intermediates in the tricarboxylic acid cycle—were upregulated, suggesting a disruption in cellular energy metabolism in OA and reflecting metabolic reprogramming of chondrocytes in an inflammatory environment (Mobasheri et al., 2017; Ge et al., 2025). Furthermore, the upregulation of methyl acetoacetate further supports the potential role of acetoacetate-related metabolites in OA inflammation (Gao et al., 2020). This study found that various metabolites, including dipeptides such as Ala-Ile, luteolin, N-acetyl-D-galactosamine 4-sulfate and 8Z,11Z,14Z-eicosatrienoic acid, were significantly downregulated in the OA group. The reduction in dipeptides suggests a decline in protein nutrient supply and absorption capacity, leading to a shortage of raw materials for cartilage repair (Rushing et al., 2022). Disruption of the anti-inflammatory fatty acid precursor 8Z,11Z,14Z-eicosatrienoic acid further exacerbates the state of low-grade inflammation (Mustonen and Nieminen, 2023). The reduction in luteolin and indole-3-carboxylic acid reflects a weakened endogenous anti-inflammatory barrier (Wang et al., 2026; Li et al., 2024); the downregulation of N-acetyl-D-galactosamine 4-sulfate indirectly indicates an imbalance between chondral matrix synthesis and degradation. It is worth mentioning that this study found that indole metabolites with anti-inflammatory activity—such as indole, indole-3-acetic acid, indole-3-lactic acid, and indole-2-carboxylic acid—tend to be upregulated in patients with OA. Previous studies have shown that these metabolites, as ligands for the aryl hydrocarbon receptor (AhR), exert anti-inflammatory and chondroprotective effects by activating the AhR signalling pathway (Wu et al., 2026). This seemingly contradictory phenomenon may reflect a compensatory immune response in which the host attempts to suppress NF-κB-mediated inflammation and counteract disease progression by upregulating AhR pathway activity (Brahmachary et al., 2026). This finding suggests that the pathophysiological mechanism of OA is not simply a single deficiency of protective factors, but more likely involves an imbalance in a complex immune regulatory network. The systemic reduction in these protective metabolites, together with the concurrent upregulation of certain compensatory indole metabolites, provides new molecular insights into the pathogenesis of OA, highlighting a state of immune imbalance rather than a straightforward loss of protective factors. In this study, we also conducted a functional prediction analysis of the gut microbiota and found that in the OA group, pathways involved in glycan biosynthesis and metabolism, as well as transport and catabolism, were all upregulated. This functional change was corroborated by specific microbiota-metabolite associations: an increase in *Bacteroidetes* abundance was positively correlated with 2-acetylglutamate levels, while a decrease in *Actinobacteria* abundance was positively correlated with downregulation of indole-3-carboxylic acid, the latter of which may have triggered a compensatory upregulation of glycan biosynthesis and metabolism. However, we did not identify any specific metabolites or microbial associations that directly correspond to the downregulation of membrane transport pathways; the biological significance of this change requires further investigation.

Subsequently, metabolic pathway enrichment analysis revealed significant alterations in multiple key pathways in the fecal metabolome of OA patients. The enrichment of the TCA cycle and related amino acid metabolic pathways correlated with the accumulation of intermediates such as 2-oxoglutaric acid and DL-malic acid, suggesting that energy metabolism in OA is reprogrammed in response to chronic inflammation, which may exacerbate oxidative stress. Alterations in phenylalanine metabolism and lysine degradation were consistent with the upregulation of phenylpyruvic acid and 4-methyl-2-oxopentanoic acid and the downregulation of L-lysine, reflecting systemic disruption of amino acid metabolism (Ge et al., 2025). The upregulation of arachidonic acid metabolism contrasts with the downregulation of 8Z,11Z,14Z-eicosatrienoic acid, suggesting an imbalance in lipid metabolism toward a pro-inflammatory state. Alterations in pyrimidine metabolism are associated with the downregulation of 3-ureidopropionic acid and a reduction in *Clostridiales*, indicating impaired pyrimidine degradation driven by the microbiota. The enrichment of glutathione metabolism, aminoacyl-tRNA biosynthesis, and biotin metabolism reflects redox imbalance, a shortage of protein synthesis substrates, and disruptions in the microbiota’s vitamin synthesis capacity, respectively. Further hierarchical analysis of upregulated and downregulated metabolites revealed that beta-alanine metabolism and phenylalanine metabolism were significantly enriched in the upregulated pathways, while pyrimidine metabolism was significantly enriched in the downregulated pathways. Furthermore, machine learning analysis suggests that N-acetyl-glutamine and Leu-Gly-Gly are potential core biomarkers. Notably, N-acetyl-glutamine is a key downregulated marker; studies have shown that N-acetyl-glutamine is significantly downregulated in the feces of IBD patients (Han et al., 2026), consistent with the findings of this study. Given the chronic inflammatory state of OA and the fact that isorhamnetin is a potential marker of upregulation, this may represent a compensatory protective response (Zhou et al., 2019). The synergistic alterations in the aforementioned metabolic pathways, combined with the directional dysregulation of key metabolites, collectively paint a pathological picture of the “gut-joint axis” in OA, characterized by the coexistence of protective metabolic collapse and the activation of pro-inflammatory pathways.

In our study, we conducted further correlation analyses to determine the relationship between gut bacteria and faecal metabolites. A reduction in beneficial bacteria such as *Actinobacteria*, *Bacilli*, *Lactobacillales*, and *Clostridiales* compromises gut health, dysregulates the immune system, and exacerbates inflammation. Notably, the decline in these beneficial bacterial populations was significantly positively correlated with the reduction in the key metabolites they drive. The decrease in *Actinobacteria* was positively correlated with the downregulation of the tryptophan metabolite indole-3-carboxylic acid (Li et al., 2024), whilst the decline in *Bacilli* and *Lactobacillales* occurred in tandem with the downregulation of luteolin—a compound with established anti-inflammatory activity, and the tryptophan derivative D-tryptophan (Wang et al., 2024, Wang et al., 2026); the reduction in *Clostridiales* was positively correlated with the decrease in isoleucylisoleucine. Given that higher genetically predicted isoleucine levels are associated with lower OA risk (Mendelian randomization), this correlation underscores the synergistic interplay between gut dysbiosis and metabolic disturbance in OA (Cui et al., 2023). This synchronous dysregulation of the ‘microbiota-metabolite’ axis collectively reveals a systemic collapse of the metabolic function of the gut’s protective microbiota across multiple functional dimensions, including amino acid synthesis, tryptophan metabolism and pyrimidine degradation. This dual loss of beneficial bacteria and their metabolites may further exacerbate the systemic state of low-grade inflammation in OA by weakening the intestinal barrier and reducing endogenous anti-inflammatory factors. It is worth noting that the elevated abundance of *Bacteroidaceae* in patients with OA was significantly positively correlated with similarly elevated levels of 2-oxoglutarate in fecal samples; this metabolite is an intermediate product of metabolic pathways such as the TCA cycle, which may suggest a compensatory adaptation by the gut microbiota under pathological conditions associated with OA (Schofield et al., 2018). Furthermore, we found that the correlation between *Bacteroidaceae* and 1-aminocyclopropane-1-carboxylate (ACC) was significant only in the NEG mode but not in the POS mode, and the response trends of ACC were opposite in the two modes. These differences may stem primarily from technical rather than biological factors, such as mode-dependent ionization efficiency, matrix effects, and the formation of different adducts ([M+H]^+^ and [M−H]^–^), as well as the fact that ACC exhibits mixed-ion characteristics (Liigand et al., 2017).

This study nevertheless has certain limitations. Firstly, the sample size was relatively small, and the findings have not been replicated in an independent, large-scale cohort or external validation cohort; therefore, the generalisability of the results requires confirmation through larger-scale, multicentre studies. Secondly, the study did not collect or report data on the participants’ use of pain relievers, anti-inflammatory medications, etc, and only 23 nutrients were included in the analysis; consequently, its ability to elucidate overall dietary patterns is limited. Future studies should account for medication use and include a more comprehensive range of nutrients to provide a more complete understanding of the impact of gut microbiota and fecal metabolites on the onset and progression of osteoarthritis.

In summary, this study validated patterns of gut microbiota dysbiosis and metabolic reprogramming in osteoarthritis from a multi-omics integration perspective, revealed specific functional couplings between microbes and metabolites, and identified potential biomarkers for early diagnosis through cross-centre machine learning. Furthermore, these differentially expressed metabolites hold potential as biomarkers for early diagnosis or therapeutic targets, laying the foundation for the development of innovative therapies to enhance bone health in middle-aged populations.

## Data Availability

The datasets presented in this study can be found in online repositories. The names of the repository/repositories and accession number(s) can be found in the article/**Supplementary Material**.
