# Supplementary material for: Fecal microbiota and metabolite profiles in patients with osteoarthritis: a cross-sectional study
Source: Front Cell Infect Microbiol. 2026 Aug 31;16:1816705. doi: 10.3389/fcimb.2026.1816705 (PMC13572143; doi:10.3389/fcimb.2026.1816705)
Supplement: Supplementary file 1 [file SupplementaryFile1.docx]

(1) **Page1：Figure S1** NMDS ordination plots comparing beta diversity between the OA and HC groups based on three distance metrics (Bray–Curtis, weighted UniFrac, and Jaccard)
(2) **Page1：Figure S2** Principal Coordinate Analysis based on Bray-Curtis and Jaccard dissimilarities
(3) **Page2：Figure S3** Differences in species composition at the family level between the two gut microbiota groups（A）；Differences in species composition at the order level between the two gut microbiota groups（B）；Differences in species composition at the class level between the two gut microbiota groups（C）
(4) **Page3：Figure S4** Significant differences in bacterial predicted functions between the two groups were identified by PICRUSt2
(5) **Page4：Figure S5** LDA map of LEfSe analysis for different species classification of intestinal flora
(6) **Page5：Figure S6** Principal Component Analysis (PCA) and OPLS-DA Analysis Score Plots in Metabolomics Positive and Negative Ion Modes.(A)PCA POS;(B)PCA NEG;(C)OPLS-DA POS;(D)OPLS-DA NEG
(7) **Page6：Figure S7** Heatmap of Correlated Metabolites in POS mode(A)；Heatmap of Correlated Metabolites in NEG mode(B)
(8) **Page7：Figure S8** Metabolic pathway diagrams of differential metabolites in positive and negative ion modes. Upregulated metabolites in POS mode(A) ;Downregulated metabolites in POS mode(B);Upregulated metabolites in NEG mode(C). Downregulated metabolites in NEG mode(D)
(9) **Page8：Figure S9** Heatmaps showing the correlations between differentially expressed metabolites and gut microbiota at various taxonomic levels (family, order, class) under cation mode (A) and anion mode (B)
(10) **Page9：Figure S10** Identification and importance ranking of key differential metabolites by random forest model, and ROC curve analysis for predictive performance.Feature importance ranking of key upregulated metabolites in POS mode (A); Feature importance ranking of key upregulated differential metabolites in NEG mode(B);ROC curves for the POS-derived/NEG-derived metabolite panel in the discovery and validation sets (AUC with 95% CI)(C/D)
(11) **Page10：Figure S11** Identification and importance ranking of key differential metabolites by random forest model, and ROC curve analysis for predictive performance.Feature importance ranking of key downregulated metabolites in POS mode (A); Feature importance ranking of key downregulated differential metabolites in NEG mode(B);ROC curves for the POS-derived/NEG-derived metabolite panel in the discovery and validation sets (AUC with 95% CI)(C/D)

**（1）**

| **(A) Bray–Curtis** | **(B) Weighted UniFrac** | **(C) Jaccard** |
| --- | --- | --- |
| Figure S1 NMDS ordination plots comparing beta diversity between the OA and HC groups based on three distance metrics (Bray–Curtis, weighted UniFrac, and Jaccard) | | |

**（2）**

| **(A) Bray–Curtis** | **(B) Jaccard** |
| --- | --- |
| Figure S2 Principal Coordinate Analysis based on Bray-Curtis and Jaccard dissimilarities | |

**（3）**

**A**

**B**

**C**

Figure S3 Differences in species composition at the family level between the two gut microbiota groups（A）；Differences in species composition at the order level between the two gut microbiota groups（B）；Differences in species composition at the class level between the two gut microbiota groups（C）

**（4）**

Figure S4 Significant differences in bacterial predicted functions between the two groups were identified by PICRUSt2

**（5）**

Figure S5 LDA map of LEfSe analysis for different species classification of intestinal flora

**（6）**

Figure S6 Principal Component Analysis (PCA) and OPLS-DA Analysis Score Plots in Metabolomics Positive and Negative Ion Modes.(A)PCA POS;(B)PCA NEG;(C)OPLS-DA POS;(D)OPLS-DA NEG

**（7）**

**A**

**B**

Figure S7 Heatmap of Correlated Metabolites in POS mode(A)；Heatmap of Correlated Metabolites in NEG mode(B)

**（8）**

| **A** | **B** |
| --- | --- |
| **C** | **D** |
| Figure S8 Metabolic pathway diagrams of differential metabolites in positive and negative ion modes. Upregulated metabolites in POS mode(A) ;Downregulated metabolites in POS mode(B);Upregulated metabolites in NEG mode(C). Downregulated metabolites in NEG mode(D) | |

**（9）**

| **A** | **B** |
| --- | --- |
| Figure S9 Heatmaps showing the correlations between differentially expressed metabolites and gut microbiota at various taxonomic levels (family, order, class) under POS mode (A) and NEG mode (B) | |

**（10）**

| **A** | **C** |
| --- | --- |
| **B** | **D** |
| Figure S10 Identification and importance ranking of key differential metabolites by random forest model, and ROC curve analysis for predictive performance.Feature importance ranking of key upregulated metabolites in POS mode (A); Feature importance ranking of key upregulated differential metabolites in NEG mode(B);ROC curves for the POS-derived/NEG-derived metabolite panel in the discovery and validation sets (AUC with 95% CI)(C/D) | |

**（11）**

| **A** | **C** |
| --- | --- |
| **B** | **D** |
| FigureS11 Identification and importance ranking of key differential metabolites by random forest model, and ROC curve analysis for predictive performance.Feature importance ranking of key downregulated metabolites in POS mode (A); Feature importance ranking of key downregulated differential metabolites in NEG mode(B);ROC curves for the POS-derived/NEG-derived metabolite panel in the discovery and validation sets (AUC with 95% CI)(C/D) | |
