# Supplementary material for: Fecal microbiota and metabolite profiles in patients with osteoarthritis: a cross-sectional study
Source: Front Cell Infect Microbiol. 2026 Aug 31;16:1816705. doi: 10.3389/fcimb.2026.1816705 (PMC13572143; doi:10.3389/fcimb.2026.1816705)
Supplement: Supplementary file 2 [file SupplementaryFile2.docx]

1. **Page2：TableS1** Comparison of Dietary Nutrient Intake Between the OA Case Group and Control Group
2. **Page3：Table S2** Statistical analysis of up‑regulated and down‑regulated metabolites in POS mode
3. **Page4：Table S3** Statistical analysis of up‑regulated and down‑regulated metabolites in NEG mode
4. **Page5：Table S4** Correlations between gut bacterial taxa with increased/decreased abundance and differentially expressed metabolites in OA

**TableS1Comparison of Dietary Nutrient Intake**

**Between the OA Case Group and Control Group**

| Nutrient | Osteoarthritis patients  (mean ± SD) | Healthy controls  (mean ± SD) | *P*-value |
| --- | --- | --- | --- |
| Water (g/d) | 1457±260 | 1425±240 | 0.599 |
| Energy (kcal/d) | 1759±290 | 1809±283 | 0.393 |
| Protein (g/d) | 65±14 | 63±14 | 0.396 |
| Fat (g/d) | 64±14 | 60±15 | 0.267 |
| Carbohydrate (g/d) | 240±48 | 231±43 | 0.239 |
| Dietary fiber (g/d) | 17±5 | 16±5 | 0.474 |
| Carotene (μg/d) | 1841±455 | 1763±441 | 0.367 |
| Retinol (μg/d) | 252±57 | 257±61 | 0.562 |
| Thiamine (mg/d) | 1±0 | 1±0 | 0.150 |
| Riboflavin (mg/d) | 1±0 | 1±0 | 0.588 |
| Niacin (mg/d) | 15±2 | 15±2 | 0.247 |
| Vitamin C (mg/d) | 105±23 | 104±22 | 0.718 |
| Vitamin E (mg/d) | 17±3 | 16±3 | 0.689 |
| Calcium (mg/d) | 718±102 | 717±104 | 0.976 |
| Phosphorus (mg/d) | 1081±107 | 1111±106 | 0.138 |
| Potassium (mg/d) | 2138±297 | 2161±292 | 0.756 |
| Sodium (mg/d) | 2396±763 | 2546±721 | 0.283 |
| Magnesium (mg/d) | 212±38 | 217±35 | 0.458 |
| Iron (mg/d) | 17±4 | 16±4 | 0.503 |
| Zinc (mg/d) | 9±2 | 8±2 | 0.333 |
| Selenium (μg/d) | 51±18 | 50±15 | 0.756 |
| Copper (mg/d) | 1±0 | 1±0 | 0.370 |
| Manganese (mg/d) | 2±0 | 2±0 | 0.459 |

**Table S2 Statistical analysis of up‑regulated and down‑regulated metabolites in POS mode**

| **Name** | **log_2_FC** | **VIP** | ***P*_value** | **FDR** | **Status** |
| --- | --- | --- | --- | --- | --- |
| 2,8-Quinolinediol | -2.491363768 | 2.136871998 | 2.94083E-06 | 4.11717E-05 | up |
| Isorhamnetin | -2.556130898 | 1.726714477 | 1.42396E-05 | 9.96774E-05 | up |
| MALTITOL | -0.56877184 | 2.017963387 | 0.001326132 | 0.006065364 | up |
| 4-Hydroxybenzaldehyde | -0.446588078 | 2.081226979 | 0.001732961 | 0.006065364 | up |
| Salsolinol | -0.811721635 | 2.200112446 | 0.003463283 | 0.008378944 | up |
| Thymine | -0.48745181 | 1.827173989 | 0.004111089 | 0.008378944 | up |
| 5-Hydroxyindole | -2.031945659 | 2.09305135 | 0.004189472 | 0.008378944 | up |
| 2-Hydroxyphenylalanine | -0.422788629 | 1.763946457 | 0.006766969 | 0.011842196 | up |
| Hypoxanthine | -0.291925172 | 1.87254672 | 0.015471546 | 0.021660165 | up |
| 7-Oxocholesterol | -0.434479847 | 1.516315689 | 0.015471546 | 0.021660165 | up |
| Xanthine | -0.289121498 | 1.63444202 | 0.019107202 | 0.023386213 | up |
| β-Alanine | -0.261407367 | 1.819485688 | 0.020045325 | 0.023386213 | up |
| Pyridine | -0.100139565 | 1.634860351 | 0.023837466 | 0.025671117 | up |
| Sphingosine | -0.705904345 | 1.598827261 | 0.047842359 | 0.047842359 | up |
| N-Acetyl-glutamine | 2.251320306 | 2.333399338 | 9.06862E-08 | 2.90196E-06 | down |
| Oxypurinol | 1.216387841 | 2.311434614 | 1.78339E-06 | 2.79111E-05 | down |
| Glycylproline | 0.575292326 | 3.176808539 | 2.61666E-06 | 2.79111E-05 | down |
| 2-PHENYLACETAMIDE | 0.431004363 | 2.830486035 | 1.50349E-05 | 0.000120279 | down |
| Prolylleucine | 0.53249301 | 1.764728731 | 6.84977E-05 | 0.000438385 | down |
| Luteolin | 1.58229454 | 1.862727082 | 9.24082E-05 | 0.000454924 | down |
| L-Pyroglutamic acid | 0.643372289 | 2.679631726 | 9.95146E-05 | 0.000454924 | down |
| 1-AMINOCYCLOPROPANE-1-CARBOXYLATE | 0.422075328 | 2.272108817 | 0.000146977 | 0.000587909 | down |
| 2-Hydroxyhippuric acid | 0.506030464 | 2.513055926 | 0.00016573 | 0.000589263 | down |
| L-Arginine | 0.462155621 | 1.345741016 | 0.000342401 | 0.001095684 | down |
| 1-O-Hexadecyl-sn-glycero-3-phosphocholine | 1.114926791 | 1.42613062 | 0.000627366 | 0.001825064 | down |
| Indoline | 0.570084581 | 1.891057407 | 0.000814672 | 0.002118108 | down |
| L-Lysine | 0.547284298 | 2.330617582 | 0.000907065 | 0.002118108 | down |
| D-Pipecolinic acid | 0.570688586 | 1.807055845 | 0.000926672 | 0.002118108 | down |
| L-Histidine | 0.315233333 | 1.433953156 | 0.001009091 | 0.002152727 | down |
| L-Tryptophan | 0.686816155 | 1.660013588 | 0.001841861 | 0.003683721 | down |
| N-METHYLTYRAMINE | 1.458208288 | 2.445074076 | 0.001957016 | 0.003683795 | down |
| Histamine | 0.430387207 | 1.25435004 | 0.002486813 | 0.004421001 | down |
| Methyl-3-hydroxybutyric acid | 0.101360656 | 1.912331836 | 0.003084571 | 0.005195067 | down |
| Cotinine | 0.598675841 | 2.113027594 | 0.005051205 | 0.007840982 | down |
| Guanine | 0.450468477 | 1.06891106 | 0.005145644 | 0.007840982 | down |
| Malic acid | 0.565930111 | 1.180377524 | 0.007670202 | 0.011156658 | down |
| 3-(2-Hydroxyethyl)indole | 0.452215221 | 1.485306295 | 0.010152182 | 0.014124775 | down |
| 3-Methylcrotonylglycine | 0.435761119 | 1.317503365 | 0.014969959 | 0.019041903 | down |
| Prolinamide | 0.217242028 | 1.842721937 | 0.015218933 | 0.019041903 | down |
| Purine | 4.206935809 | 1.125989799 | 0.015471546 | 0.019041903 | down |
| 2-Phenylglycine | 0.549179255 | 2.106007319 | 0.017066133 | 0.020056682 | down |
| Hexadecanamide | 0.126567838 | 1.47223643 | 0.017628813 | 0.020056682 | down |
| 4-Pyridoxic acid | 0.373398471 | 1.599543713 | 0.018207688 | 0.020056682 | down |
| L-Dopa | 0.317144689 | 1.223659544 | 0.01880314 | 0.020056682 | down |
| Quercetin | 2.166105892 | 1.492274464 | 0.030919254 | 0.031916649 | down |
| 2-Methylnicotinamide | 0.307656987 | 1.340586834 | 0.047172945 | 0.047172945 | down |

Note: False discovery rate (FDR) correction was performed using the Benjamini–Hochberg (BH) method.

**Table S3** **Statistical analysis of up‑regulated and down‑regulated metabolites in NEG mode**

| **Name** | **log_2_FC** | **VIP** | ***P*_value** | **FDR** | **Status** |
| --- | --- | --- | --- | --- | --- |
| Indole-2-carboxylic acid | -2.485689547 | 2.058092436 | 1.76825E-05 | 0.000795714 | up |
| Isorhamnetin | -2.606514055 | 1.91521933 | 5.1833E-05 | 0.001166243 | up |
| 9-Methoxycarbonyldec-9-enoic acid | -1.558093047 | 1.852373955 | 0.000124056 | 0.001860841 | up |
| 2-Oxoglutaric acid | -1.322049213 | 2.776395726 | 0.000655507 | 0.006719901 | up |
| 3-METHYL-2-OXINDOLE | -1.245341902 | 1.609295732 | 0.000763509 | 0.006719901 | up |
| Indole | -0.428134438 | 1.310113647 | 0.000926672 | 0.006719901 | up |
| N-Acetylasparagine | -0.579162202 | 1.273123928 | 0.001052798 | 0.006719901 | up |
| Xanthurenic acid | -2.859608768 | 1.679856609 | 0.001194649 | 0.006719901 | up |
| 1-AMINOCYCLOPROPANE-1-CARBOXYLATE | -0.586256917 | 1.857037075 | 0.001697998 | 0.007794919 | up |
| FA 18:4+2O | -0.910386474 | 2.311536979 | 0.001841861 | 0.007794919 | up |
| N-Acetyl-D-alloisoleucine | -0.802928362 | 1.918958684 | 0.001957016 | 0.007794919 | up |
| Phenylpyruvic acid | -0.690111111 | 2.245360588 | 0.00216372 | 0.007794919 | up |
| FA 16:3 | -1.172737165 | 1.93308056 | 0.002251866 | 0.007794919 | up |
| β-Estradiol-17β-glucuronide | -0.821877599 | 2.165598557 | 0.002909737 | 0.009075769 | up |
| 3-Methylglutaric acid | -0.786844157 | 1.448209515 | 0.003025256 | 0.009075769 | up |
| 4-METHYL-2-OXO-PENTANOIC ACID | -0.66029597 | 1.952032589 | 0.005145644 | 0.014472125 | up |
| N-Acetylglutamic acid | -0.473000604 | 1.60521052 | 0.006070135 | 0.0157372 | up |
| DL-Malic acid | -0.436377581 | 2.156803827 | 0.00629488 | 0.0157372 | up |
| Imidazoleacetic acid | -0.432571866 | 1.84981963 | 0.007401703 | 0.017530348 | up |
| Capryloylglycine | -0.81271024 | 1.89600369 | 0.008680034 | 0.01926343 | up |
| Methyl acetoacetate | -0.500023146 | 1.746789374 | 0.008989601 | 0.01926343 | up |
| 4-Acetamidobutanoic acid | -0.425145083 | 1.519068409 | 0.009978297 | 0.020410153 | up |
| O-Acetylserine | -0.302691423 | 1.444288332 | 0.010875007 | 0.021277187 | up |
| Indole-3-acetic acid | -1.050904564 | 2.182057937 | 0.01245919 | 0.023360982 | up |
| Succinic semialdehyde | -0.509836535 | 2.309017809 | 0.014244434 | 0.025639982 | up |
| Hexanoic acid | -0.507468715 | 1.302283865 | 0.015218933 | 0.026340461 | up |
| 2-KETO-3-DEOXY-D-GLUCONIC ACID | -0.420216518 | 1.284560049 | 0.017628813 | 0.029381355 | up |
| gamma-Glutamylmethionine | -1.178004773 | 1.14648721 | 0.019728245 | 0.031706109 | up |
| Indole-3-lactic acid | -1.947510269 | 1.449511599 | 0.02135854 | 0.033142563 | up |
| Uracil | -0.240113093 | 1.715065572 | 0.022746053 | 0.033521437 | up |
| Hypoxanthine | -0.252041833 | 1.882062127 | 0.023468719 | 0.033521437 | up |
| 4-Oxoproline | -0.319871117 | 1.37346418 | 0.023837466 | 0.033521437 | up |
| Ferulic acid | -1.414467824 | 1.577113497 | 0.026562211 | 0.035699072 | up |
| D-Salicin | -1.20342067 | 1.271611147 | 0.026972632 | 0.035699072 | up |
| gamma-Glutamylleucine | -0.456768437 | 1.423293649 | 0.030001799 | 0.037042977 | up |
| TYRAMINE | -0.418681942 | 1.508220468 | 0.030001799 | 0.037042977 | up |
| Xanthine | -0.284341253 | 1.574264459 | 0.030457559 | 0.037042977 | up |
| 2-Methoxy-4-vinylphenol | -0.771036827 | 1.223837375 | 0.03186069 | 0.037729764 | up |
| 4-Hydroxybenzaldehyde | -0.36000211 | 1.017848547 | 0.034322418 | 0.03960279 | up |
| Pantothenic acid | -0.343023505 | 1.693531711 | 0.035351673 | 0.039770632 | up |
| Tretinoin | -4.311561063 | 1.233173802 | 0.0403153 | 0.0442485 | up |
| 3,3-Dimethylglutaric acid | -0.591939008 | 1.208571117 | 0.044573552 | 0.046511428 | up |
| D-2-Hydroxyglutaric acid | -0.613178137 | 1.491332896 | 0.0452118 | 0.046511428 | up |
| dihydroferulic acid | -1.492151229 | 1.487662606 | 0.046511428 | 0.046511428 | up |
| L-Glutamic acid | -0.277799688 | 1.191516336 | 0.046511428 | 0.046511428 | up |
| Leu-Gly-Gly | 0.748908413 | 1.988599012 | 5.14891E-08 | 1.48402E-06 | down |
| Ala-Ile | 0.902243021 | 2.535866834 | 9.37272E-08 | 1.48402E-06 | down |
| Isoleucylisoleucine | 0.745654668 | 1.931298232 | 9.68669E-08 | 1.48402E-06 | down |
| Alanyltyrosine | 0.717354671 | 1.734598017 | 1.14156E-07 | 1.48402E-06 | down |
| 2'-Deoxyuridine | 0.966706477 | 2.282334997 | 1.5813E-07 | 1.64455E-06 | down |
| Thymidine | 1.075577156 | 2.87642198 | 2.32726E-07 | 2.01696E-06 | down |
| Adenosine | 1.434726803 | 2.420566727 | 4.24765E-07 | 3.15539E-06 | down |
| 6-Hydroxymelatonin | 0.572139257 | 1.514842023 | 1.07077E-06 | 6.95998E-06 | down |
| Luteolin | 2.003676045 | 2.064819486 | 2.06816E-06 | 1.19494E-05 | down |
| Ala-Phe | 0.735065682 | 1.947663985 | 9.1738E-06 | 4.77037E-05 | down |
| Guanosine | 0.660639964 | 1.016434323 | 1.96884E-05 | 9.30724E-05 | down |
| 2,3-Dinor-11β-prostaglandin F2α | 1.63140259 | 1.942779754 | 2.37303E-05 | 0.000102831 | down |
| 6-PHOSPHOGLUCONIC ACID | 1.423416887 | 1.06744778 | 0.000112517 | 0.000450068 | down |
| gamma-Glutamylglutamine | 0.701859169 | 2.63049476 | 0.000130236 | 0.000483732 | down |
| 4-HYDROXYCYCLOHEXANECARBOXYLIC ACID | 2.297204463 | 1.893478595 | 0.000154225 | 0.000534647 | down |
| Glycyl-L-leucine | 0.641149318 | 2.123695693 | 0.000242 | 0.000740234 | down |
| 3-Ureidopropionic acid | 0.342883026 | 1.057251299 | 0.000242 | 0.000740234 | down |
| FA 13:3+1O | 0.767297 | 1.188030467 | 0.000305254 | 0.000881846 | down |
| Uridine | 0.909610153 | 2.091587475 | 0.000375119 | 0.001026643 | down |
| Indole-3-carboxylic acid | 1.851744593 | 1.444446914 | 0.000410742 | 0.00106793 | down |
| 4-Phenylbutyric acid | 1.596979538 | 1.846100517 | 0.000670013 | 0.00165437 | down |
| 1,7-Dimethyluric acid | 1.161698261 | 1.736076904 | 0.000699926 | 0.00165437 | down |
| Tyrosol | 1.613772506 | 1.502951621 | 0.000763509 | 0.001726194 | down |
| Caprylic acid | 0.688430852 | 1.865572472 | 0.000967069 | 0.002095315 | down |
| Catechin | 2.109177618 | 2.127373321 | 0.001663685 | 0.003460464 | down |
| 5-Hydroxylysine | 2.092213646 | 2.27860812 | 0.001879535 | 0.00375907 | down |
| HOMOVANILLIC ACID SULFATE | 2.37799763 | 1.592778623 | 0.001996846 | 0.003845778 | down |
| Guanine | 0.710126764 | 1.257845563 | 0.00216372 | 0.004018337 | down |
| N-Fructosyl pyroglutamate | 0.641250855 | 1.822046943 | 0.003144944 | 0.005639211 | down |
| Asp-Glu | 0.464522245 | 1.375161492 | 0.00359863 | 0.006192367 | down |
| Adenine | 0.797341527 | 1.094115358 | 0.003738772 | 0.006192367 | down |
| Perillic acid | 2.187882768 | 1.317962636 | 0.003810687 | 0.006192367 | down |
| Glu-Thr | 0.433488664 | 1.638902628 | 0.004189472 | 0.006601593 | down |
| Epigallocatechin | 2.3176957 | 1.068152953 | 0.004958336 | 0.007583338 | down |
| Gallic acid | 3.152996912 | 1.951373275 | 0.005539563 | 0.008230209 | down |
| Methoxytyrosine | 0.61365265 | 1.935260706 | 0.006070135 | 0.008767973 | down |
| Quinic acid | 1.286385048 | 1.333136622 | 0.008528852 | 0.011986495 | down |
| N-Acetylglycine | 0.217403775 | 1.096083266 | 0.010152182 | 0.01389246 | down |
| OPHTHALMIC ACID | 0.447255323 | 1.65947533 | 0.013104004 | 0.017472005 | down |
| Quercetin | 1.940713521 | 1.682979856 | 0.014244434 | 0.018066112 | down |
| L-Arginine | 0.777359105 | 1.349338574 | 0.014244434 | 0.018066112 | down |
| 3-Hydroxybutyric acid | 0.765145482 | 2.240067283 | 0.014969959 | 0.018534234 | down |
| 2-Hydroxy-3-methylbutyric acid | 0.375884516 | 1.167521869 | 0.017916203 | 0.021666106 | down |
| 5-Acetylamino-6-amino-3-methyluracil | 1.622196016 | 1.464304295 | 0.023837466 | 0.027369215 | down |
| N-Acetyl-D-galactosamine 4-sulfate | 1.246102821 | 1.75345091 | 0.024211229 | 0.027369215 | down |
| N-Acetylalanine | 0.47108037 | 1.185741174 | 0.024211229 | 0.027369215 | down |
| Caffeic acid | 1.515287822 | 1.647352474 | 0.024974019 | 0.027630829 | down |
| Homogentisic acid | 0.560611716 | 1.613417163 | 0.030919254 | 0.033495858 | down |
| 8Z,11Z,14Z-Eicosatrienoic acid | 0.66394202 | 1.735150789 | 0.032340553 | 0.034320587 | down |
| D-Tryptophan | 1.013029641 | 1.092847455 | 0.033318879 | 0.034651634 | down |
| Arachidonic acid | 0.314615375 | 1.297639199 | 0.038598839 | 0.039355679 | down |
| ALLANTOIN | 1.237504103 | 1.02620467 | 0.0452118 | 0.045211800 | down |

Note: False discovery rate (FDR) correction was performed using the Benjamini–Hochberg (BH) method.

**Table S4 Correlations between gut bacterial taxa with increased/decreased abundance and differentially expressed metabolites in OA**

| **Metabolites** | **Microbes** | **estimate** | ***p*.value** | ***p*.adjust** |
| --- | --- | --- | --- | --- |
| 2-Oxoglutaric acid | *Bacteroidaceae* | 0.46339968 | 4.74411E-07 | 2.79945E-05 |
| Methyl acetoacetate | *Bacteroidaceae* | 0.454969231 | 7.98705E-07 | 4.24685E-05 |
| Phenylpyruvic acid | *Bacteroidaceae* | 0.454888082 | 8.0267E-07 | 4.24685E-05 |
| 4-METHYL-2-OXO-PENTANOIC ACID | *Bacteroidaceae* | 0.445970741 | 1.37305E-06 | 6.52339E-05 |
| Indole | *Bacteroidaceae* | 0.395018371 | 2.27915E-05 | 0.00060294 |
| 4-Hydroxybenzaldehyde | *Bacteroidaceae* | 0.390338796 | 2.88916E-05 | 0.000717045 |
| Isoleucylisoleucine | *Clostridiales* | 0.324626378 | 0.000575629 | 0.007642251 |
| Uridine | *Clostridiales* | 0.295665307 | 0.001784264 | 0.015836803 |
| Ala-Ile | *Clostridiales* | 0.295160381 | 0.001818071 | 0.016032076 |
| 3-Ureidopropionic acid | *Clostridiales* | 0.294168564 | 0.001886176 | 0.016418358 |
| 6-Hydroxymelatonin | *Clostridiales* | 0.275098618 | 0.003735461 | 0.026698714 |
| Ala-Phe | *Clostridiales* | 0.267614904 | 0.004825353 | 0.032928791 |
| Alanyltyrosine | *Clostridiales* | 0.267317359 | 0.004874041 | 0.033094741 |
| Guanosine | *Clostridiales* | 0.26018529 | 0.006180558 | 0.038678328 |
